# The influence of leadership on stakeholder involvement in the digitalisation of the UK healthcare sector: An activity theory perspective

**DOI:** 10.1177/20552076261462656

**Published:** 2026-06-17

**Authors:** Boroto Hwabamungu, Paul Shepherd

**Affiliations:** 1 Department of Architecture and Civil Engineering, University of Bath, Bath, UK

**Keywords:** leadership, digitalisation, digital health, healthcare sector, stakeholder involvement, stakeholder trust

## Abstract

**Objective:**

Leadership plays a critical role in shaping an organisation’s digitisation journey and driving complex change processes. As the necessity to involve stakeholders in achieving digitalisation objectives across sectors is becoming increasingly evident, this study used Activity Theory as theoretical lens to explore leadership influence on stakeholder involvement in the digitalisation of the UK healthcare sector.

**Methods:**

This study followed an interpretive qualitative approach using the UK healthcare sector as a case study. Data were collected using semi-structured interviews and survey responses complementarily.

**Results:**

A thematic analysis of 14 interview transcripts and 121 survey responses revealed that leadership’s influence on stakeholder involvement transcends leadership styles and traditional attributes. It is multi-dimensional and encompasses the ability to navigate multiple systemic elements to shape stakeholders’ commitment to the common digitalisation goal.

**Conclusions:**

This study provides insights into the moderating and mediating role of leadership in stakeholder involvement, by dissecting its influence across five major dimensions: involvement integrability, commitment to stakeholder involvement, stakeholder inclusivity and engagement, leadership-digitalisation-stakeholder trust triality, and leadership obstructiveness.

## 1. Introduction

The relevance of leadership in achieving organisational goals across disciplines has been widely discussed in the literature over the past years.^1^ It is the key to achieving organisational change and successfully deploying and adopting digital technologies (DTs).^2^ It plays particularly a critical role in achieving digitalisation’s objectives in the healthcare sector,^3^ where evidence-based best practices are essential^4^ and where a context specific leadership style is necessary to effectively drive digital change.^5^ While leadership has received scholarly attention in disciplines such as social sciences, business and management, there is a need to apply leadership principles to the context of the healthcare sector.^6^ The sector’s digitalisation complexity has raised calls for further research on the implementation and adoption of DTs.^7^ Such scholarly endeavours will positively impact the sector’s digital transformation journey^8^ by providing an understanding of the sector’s intricacies.

The healthcare sector is a multiple stakeholder environment. This renders digitalisation endeavours challenging.^9–11^ Lack of stakeholder involvement, and poor stakeholder involvement practices, negatively impact organisational change.^12^ In its complexity, stakeholder involvement is affected by multi-faceted systemic elements, including leadership and stakeholder attributes.^12^ The literature reveals a lack of research on the influence of leadership on digitalisation in the healthcare sector.^13^ Moreover, leadership style change is necessary to navigate the complexities of disruptive innovations and digital transformation for effective change in the sector.^14^ More research on the influence of leadership approaches in the changing healthcare environment is critical.^15^

As efforts are continuously being made to improve healthcare service delivery in the UK using innovative DTs,^16^ and as these efforts are affected by multiple adoption hinderances,^17^ this study was undertaken to investigate the influence of leadership in stakeholder involvement, identify how this affects the digitalisation process in the UK healthcare sector, and to contribute to the development of informed guidance on leadership to improve stakeholder involvement and the adoption of digital technologies.

## 2. Background

Interest in the digitalisation and digital transformation of the healthcare sector to improve healthcare services has been increasing globally over the years.^18^ The UK government has embraced this global trend to capitalise on these innovative technologies’ promises of value creation for all the different stakeholders.^19^ While the potential benefits are diverse,^20^ adoption in the sector is slow, affected by different process, technical, regulatory, leadership and people factors.^21^ The industry’s slow pace of adoption necessitates timely research to develop a better understanding of the influence of these factors and the triple complexities of the healthcare sector, digitalisation and stakeholder involvement, as a step towards improving digitalisation outcomes. Some of these factors include: users’ digital literacy,^22^ the diversity of individuals, groups and organisations in the stakeholder landscape,^8^ different levels of adoption willingness and readiness among stakeholders,^23^ and good governance and stakeholder involvement.^21^ This is particularly important for the UK healthcare sector,^16^ where the involvement of the multiple stakeholders is a prerequisite for success.^24^

The healthcare sector is a complex environment with a broad range of stakeholders.^9,11,25^ They include: patients or users, families of patients, clinicians, nurses, administrators, employees, health institutions and organisations, researchers, payers’ organisations and authorities, healthcare policymakers, service developers, consultants, and consumer advocates.^26^ Their involvement is essential to achieving digitalisation objectives.^27^ The role of stakeholders in the attainment of different organisational objectives, and the guiding principles of stakeholder theory in this regard, are elaborated in the literature.^28^ Stakeholder theory has evolved over the years^29^ and its relevance spans disciplines^30^ and sectors.^31^ Its descriptive, instrumental and normative dimensions,^32^ and the different stakeholder classification models/typologies^28,33^ provide useful stakeholder management exploratory lens. Under the stakeholder management umbrella, notions such as stakeholder power de,^34^ stakeholder identification,^35^ stakeholder engagement,^36^ stakeholder relationships,^37^ stakeholder collaboration,^38^ and stakeholder involvement^39^ can be identified.

The criticality of leadership for stakeholder involvement, the adoption of DTs and the achievement of good organisational digitalisation outcomes cannot be overemphasised, particularly in the multi stakeholder healthcare sector environment context. Effective leadership is primarily required to manage the complexity of the healthcare sector environment.^40,41^ In such environments, good leadership is essential in ensuring good stakeholder engagement and building meaningful trust relations with all stakeholders.^42^ Hence, the emergence of the digital leadership notion for successful digital transformation in the current disruptive DTs-driven business environment.^43^ Six critical digital transformation leadership attributes have been identified in this regard: digital knowledge and literacy, vision, understanding of customers, agility, risk-taking and collaboration.^44^ In the case of AI in the healthcare sector, for example, leadership perceptions about DTs’ relevance and complexity have implications on DTs’ future deployment.^45^

The broad and rich scholarly discourse provides insight into different leadership theories and styles, including great-man theory, trait theory, contingency or situational theories, transactional theory, and transformational theory.^46^ Among these, transformational leadership has been identified as more appropriate and effective in the healthcare sector.^41^ Despite the theories’ historical origins and underlying principle variances, leadership concepts continue to evolve in practice and contexts.^47^ The concept’s multiple descriptions, sometimes incomplete, necessitate a holistic definition that underlines its etymological and influence manifestation richness.^48^ As an example, the dyadic leadership-follower relationship provides grounds for exploring the dynamics of leadership influence in different contexts,^42,49^ while the different leadership attributes and followers’ characteristics are essential to achieving the leader’s vision and change objectives.^50,51^ In this study we adopt the following leadership definition adapted from the leadership literature^48^ and its extension to the healthcare sector^52^: *Leadership is the ability to effectively and ethically influence people in their diversity to achieve an organisation’s mission and objectives in a coordinated vision-sharing-and-adherence process*.

The current DTs landscape context^53^ and global DTs trends in the healthcare sector^54^ necessitate healthcare specific leadership approaches^41,55^ and leaders who possess the necessary capabilities to navigate the complex healthcare environment.^56^ Research on leadership in the ever changing global environment^57^ and its influence on the complex digitalisation environment,^58^ is therefore essential to develop leadership insight for digitalisation in the healthcare context. To contribute in this regard, the authors investigated the influence of leadership on the involvement of stakeholders in the UK health sector. Using Activity Theory (AT) as a theoretical lens, this study investigated the following research question: what leadership dimensions influence stakeholder involvement in the UK healthcare sector? AT has been extensively used beyond its Psychology and Anthropology roots.^59–61^ Its wide use in the field of information systems research is well documented.^59,62^ The theory provides exploratory capabilities in different studies, including digital innovation,^63^ digitalisation,^64^ DTs’ use,^65^ Artificial Intelligence (AI) use in education,^66^ and digitalisation in the healthcare sector.^67,68^

**Figure 1.**
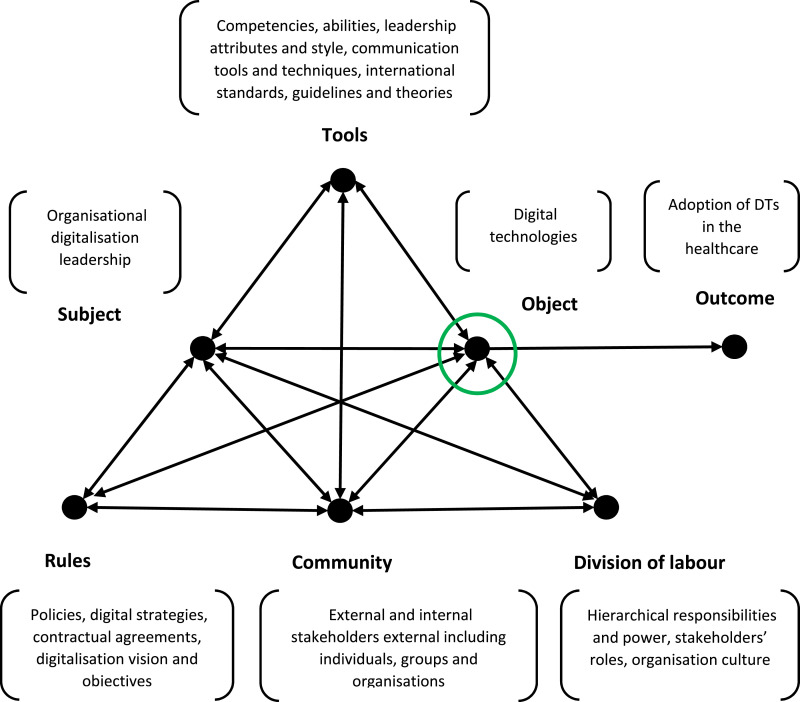
Adaptation of Engestrom’s AT model to the digitalisation of the UK healthcare sector.

AT notions of the collective, artefact-mediated object-oriented activity system as the unit of analysis, the multi-voicedness of activity systems, historicity, contradictions and expansive transformation are fundamental for the functioning of the activity system.^61^ Over the years, AT has evolved from Vygotsky’s three-dimension tool-mediated action model, to enhanced versions such as Engestrom’s extended version elements^61^ and Korpela’s Activity Analysis and Development (ActAD) framework.^60^ In recent years, scholarly efforts have been undertaken to further develop the theory in its Fourth Generation Activity Theory (4GAT) version, adapting to current changing activity dynamics.^69^ Each version provides greater exploratory capabilities emanating from further elaboration of an activity system’s elements. Engestrom’s extended AT model provides great exploratory capabilities engrained in the interaction between six activity systems elements, namely subject, object, tools, rules, community, and division of labour.^61^ In our study, the model was used as the analytic tool to understand the interplay between leadership and multiple stakeholder involvement in digitalisation and explore the interrelatedness between the digitalisation activity system elements in the UK healthcare sector as illustrated in Figure 1.

Leading digitalisation in the healthcare sector is a multi-faceted undertaking in a complex activity system involving multiple stakeholders. To explore the influence of leadership on the involvement of stakeholders, we drew from the theory’s multi-activity interaction and activity system dynamics, where one object in one activity can itself be the subject in another sub-activity within the larger digitalisation activity system.

Table 1 provides an illustration of the adaptation of activity theory to this study’s data analysis.

**Table 1.** Activity theory concepts’ adaptation to the study.

AT concepts	Definition	Adaptation to the study
Subjects	The individual or group engaged in the activity^70^	Healthcare digitalisation leadership
Objects	The goal or purpose towards which the activity is directed^70^	Digital technologies and stakeholders
Outcome	The desired results or transformation that the activity seeks to produce.^70^	Adoption of DTs in the healthcare sector of the UK.
Division of labour	The distribution of tasks, responsibilities, and roles among participants in the activity system.^70^	Hierarchical responsibilities and power, stakeholders’ roles, and organisation culture.
Tools	The instruments or resources used to transform the object into the desired outcome.^61^	Competencies, abilities, leadership attributes and style, communication tools and techniques, international standards, guidelines and theories.
Rules/norms	The norms, conventions, or guidelines that govern interactions and behaviours within the activity.^70^	Policies, digital strategies, contractual agreements, digitalisation vision and objectives.
Community	The broader social context or group within which the activity occurs.^70^	External and internal stakeholders including individuals, groups and organisations that interact within the healthcare activity system.
Contradictions	Historical tensions within the activity system that can lead to disruptions and drive change.^61^	Structural, organisational, and historically embedded contradictions that manifest at different levels and that affect stakeholder involvement.

The interaction between these elements provided insight into the dynamics of leadership influence in the complex digitalisation multiple-activity system with a particular focus on stakeholder involvement in the healthcare sector of the UK.

## 3. Methodology

This study adopted a qualitative interpretive research method for an in-depth exploration of the research phenomenon in its social and cultural context.^71^ We followed Klein and Myers^72^ seven principles of qualitative research, namely: the hermeneutic circle, contextualization, interaction between the researchers and the subjects, abstraction and generalisation, dialogical reasoning, multiple interpretations, and suspicion. This method allows the researcher to be immersed within the research phenomenon, to capture its complexities, and to assign meaning through interpretation, using methods such as case studies, action research and ethnography.^73^ This method is relevant in the current context of emerging DTs and is suited to better understand their complexities.^74^ This study followed a case study approach,^75,76^ to capture the complexities of leadership influence in stakeholder involvement in digitalisation, and to develop deep insight into their intricacies from an AT perspective.

Using UK healthcare sector digitalisation as our case study, we collected data using qualitative interviews^77^ and qualitative surveys^78^ complementarily as preferred techniques. In particular we used semi-structured interviews^71^ and online qualitative surveys^79^ for richness of data and broad participant access. Purposive sampling^80^ allowed us to collect data from 135 participants with diverse digitalisation backgrounds and roles in the UK healthcare sector. We conducted 14 Semi-structured interviews and collected 121 survey responses. The interviews were conducted between December 2023 and March 2024 with 3 digitalisation leaders, 2 nurses, 1 DTs provider, 2 digital health consultants, 2 clinicians, 3 digitalisation academics and 1 pharmaceutical industry consultant. The survey was designed using QuestionPro software^81^ and administered using Prolific between March 2024 and May 2024. Each interview lasted between 30 and 45 minutes. The survey was estimated to be completed within the same time frame. To ensure the quality of the data collected from the survey, participants were pre-screened and their responses verified to avoid duplication using the platform built-in checks. Additionally, the responses were checked manually to ensure only fully completed and answered survey responses were included in the dataset. Respondents included a mix of 80 healthcare professionals, 32 IT staff and data analysts, 4 digitalisation managers and 5 healthcare researchers. Interviewees and survey respondents had different number of years’ experience in digitalisation grouped as follow: 66 participants [1-5 years], 38 participants [6-10 years], 11 participants [11-15 years], 15 participants [16-20 years], and 5 participants [21-more years].

**Figure 2.**
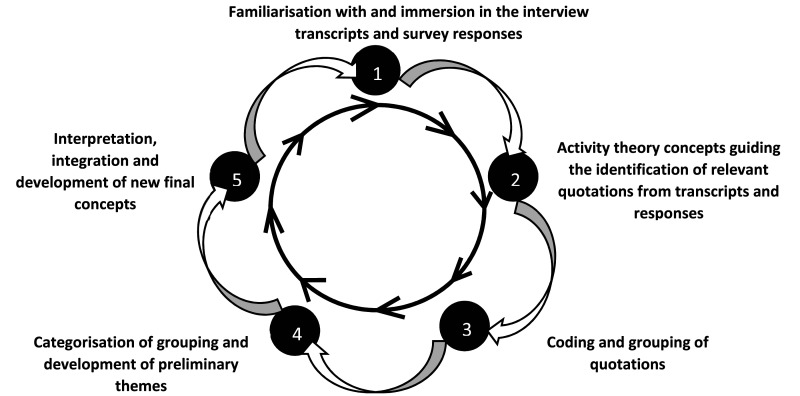
Thematic analysis process.

Using manual Thematic analysis^82^ and NVivo 14 software,^83^ data was analysed through a multiple iteration process that involved the identification of themes from interviews and survey responses from an Activity Theory perspective, grouping of these themes, continuous refinement, and inductive AT revisitation to map the manifestation of leadership’s influence in stakeholder involvement as illustrated in Figure 2.

**Stage 1. Familiarisation with and immersion in the interview transcripts and survey responses:** we systematically went through the interview transcripts and survey responses to gain a deep understanding of the participants’ experiences and perspectives, and the richness in their responses. In this exercise, researchers took analytical notes while reflecting on and making sense of existing activity system dynamics. In parallel, the transcripts and survey responses were input into NVivo 14 software.
**Stage 2. Activity theory concepts guiding the identification of relevant quotes from transcripts and responses:** using AT concepts described above in Table 1, we started to identify and select corresponding quotes both manually and using NVivo 14.
**Stage 3. Coding and grouping of quotes:** using a manually approach complemented by NVivo 14, we coded the identified quotes, organised them into clusters in a code manual.
**Stage 4. Categorisation of grouping and development of preliminary themes:** in an iterative comparative analysis process, the identified quotes and excerpts were grouped, categorises and synthesized. This process generated 78 preliminary themes.
**Stage 5. Interpretation, integration and development of new final concepts:** further iterations and categorisation of these 78 preliminary themes led to the distinction of 20 themes and the definition of 5 final integrated concepts as summarised in Table 2 below.

In this iterative process, emerging themes were continuously compared, refined and categorised. The themes emerged across both the interviews and the survey responses analysis. In this process, a comparison between the emerging themes was carried out to assess complementarity, similarities, and divergence among themes. While one researcher conducted the analysis and coding, saturation was reached when no new major themes or categories could be identified and the two researchers reached consensus that there was no further insight appearing across the interviews and survey responses data.

## 4. Results

This study has identified the following five key dimensions, capturing the manifestation of the influence of leadership on stakeholder involvement in the digitalisation of the UK healthcare sector: involvement integrability, commitment to stakeholder involvement, stakeholder inclusivity and engagement, leadership-digitalisation-stakeholder trust triality, and leadership obstructiveness as highlighted in Table 2.

**Table 2.** Thematic analysis final iteration.

Sub-themes	Final concepts
• Leadership competencies as driver of stakeholder involvement	**Involvement integrability**
• Leadership’s holistic approach to digitalisation	
• Relevance of leader’s digital and clinical experience	
• Leadership style’s effectiveness on stakeholder involvement	
• Persistence to bring stakeholders together	**Commitment to stakeholder involvement**
• Understanding of all stakeholders’ value for project success	
• Commitment to sharing and achieving digitalisation vision	
• Practicality in demonstrating digitalisation value	
• Ability to bring multiple stakeholders together	**Stakeholder inclusivity and engagement**
• Choice and use of multiple engagement tools and channels	
• Playing involvement champion role	
• Practice of participatory techniques and use of human-centeredness principles	
• Enabler of stakeholders’ trust in digitalisation	**Leadership-digitalisation-stakeholder trust triality**
• Leadership trustworthiness	
• Leadership trust in digital technologies’ relevance	
• Building trust relationship	
• Hierarchical leadership as a barrier to stakeholder involvement	**Leadership obstructiveness**
• Blockers of digitalisation leader’s actions	
• Leadership’s power and digitalisation conflicting interests	
• Leadership change and impact on stakeholder landscape	

### 4.1. Involvement integrability

As a process and an activity, stakeholder involvement is primarily dependent on how leaders can effectively integrate it into the complex digitalisation process. This integrability is critical for the development of essential stakeholder involvement strategies, including the choice of effective communication tools, techniques and platforms. Integrability is dependent on different leadership factors including style, skills, characteristics, attitude, persistence, stakeholder involvement vision, and understanding of the criticality of a holistic approach that prioritises stakeholder involvement, as reflected by the following participants:

“Leadership made informed decisions regarding technology selection, implementation planning, and integration strategies, based on input from stakeholders and alignment with organizational goals.” [**Participant 35]**

“We have recently had a new CEO take over and he's running the place like a dictatorship. He's in charge and there's no consultation with staff”. [**Participant 07]**

This integrability contributes to the achievement of benefits such as informed decision-making, shared digitalisation vision, stakeholders’ recognition of digitalisation value, and improved motivation for DTs’ adoption as highlighted by the following participant:

“Stakeholder involvement is super important…Open Institute 4.0 mentioned that if you are going to transform an industry, it’s no longer down to the performance of one organisation, it’s about the ecosystem and subsystem. I think the most important thing is shared vision, and shared values. So, there has to be a shared vision that all stakeholders buy into…” [**Participant 14]**

“It’s all down to the leader, if the leader doesn’t care, isn’t present, it’s not going to happen. If the leader cares, which is probably the number one thing, because the leader sets the culture and has been doing a great job in terms of engagements with all the clinical and non-clinical staff”. [**Participant 63]**

### 4.2. Commitment to stakeholder involvement

Effective stakeholder involvement has been identified to be dependent on how committed the leadership is to undertaking processes to operationalise the integral approach. This is a de-facto determinant for the creation of an environment that encourages engagement and participation. It is dependent on the leadership’s understanding of the relevance of stakeholder involvement in fostering a shared digitalisation vision.

“We brought them to the hospital and actually brought the piece of equipment and we demonstrated this is what the nurse does taking observations, and initially there was resistance from the chief nursing information officer who said the nurse will not accept this technology because it will make them accountable, but exactly the opposite happened… “seeing is believing” is another thing. For me it is always demonstrate and demonstrate. Don’t just talk about it, demonstrate it”. [**Participant 11]**

The leaders’ coordinated efforts, engagement strategy and use of practical communication techniques to actively involve stakeholders are essential to effectively orchestrate change acceptance and stakeholders’ commitment to the achievement of the digitalisation vision, as reflected by the following participants:

“The leaders have to work hard to engage with the staff through the process. In the engagement process the leadership has made it clear that new tech is required to move the business forward and so the staff need to embrace the changes”. [**Participant 35]**

“Our Leaders articulate the importance of digitalization, align it with organizational goals, bring all stakeholders together and create a sense of urgency and commitment among stakeholders”. [**Participant 127]**

### 4.3. Stakeholder inclusivity and engagement

Leadership enables the establishment of an extensive digitalisation stakeholder landscape by promoting inclusivity and championing stakeholder engagements. This is dependent on the leadership’s commitment to stakeholder involvement and their determination to bring all relevant stakeholders onboard. In these instances, leadership plays an engagement accelerator role conducive to stakeholders’ acceptance and technology’s timely deployment, as highlighted by the following participants’ responses:

“About the one system, I went around them, I went to the top doctors, the head of medical equipments, the head of facilities, the IT people and we just got it done. And I just told them, we did the procurement in 3 weeks, normally it takes a year, 2 years. And with another case, I couldn’t get around them, they were blocking and blocking the process”. [**Participant 04]**

“These people need to be shown proof of the benefits of digitalisation - that it is their friend, not foe. This needs to be done well-before implementation. Also getting clinical staff involved in the implementation of digitalisation projects gives them a sense of ownership - it is not just another IT project”. [**Participant 09]**

Leadership’s inclusivity efforts are critical and need to be sustained throughout the digitalisation process to minimise non-involvement and non-adoption risks as reflected by the following digitalisation consultant:

“In previous organisations they [Leaders] have presented significant challenges, by failing to recognise the value of engaging all the clinical members. In others, they [Leaders] have advocated and championed the involvement, which is usually extremely helpful, but in some instances I would need to advise leadership on their tone and approach, to avoid creating or exacerbating resistance to adoption”. [**Participant 130]**

### 4.4. The leadership-digitalisation-stakeholder trust triality

**Figure 3.**
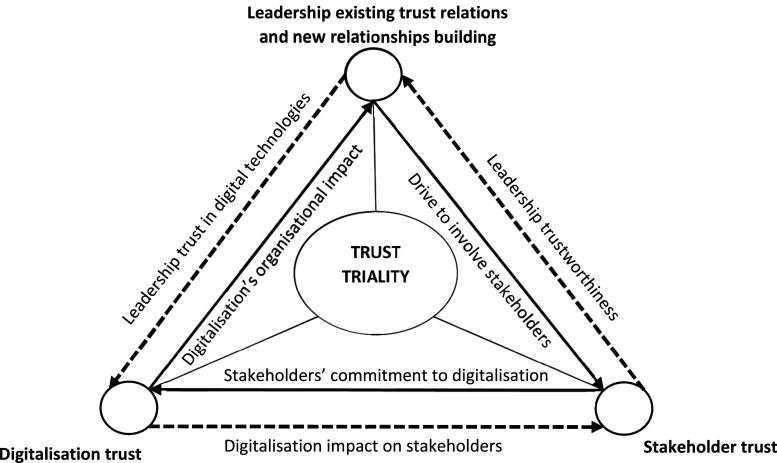
The leadership-digitalisation-stakeholder trust triality.

Leadership is a great catalyst to stakeholders’ trust in the digitalisation process. The trust relationship has a tri-dimensional influence as illustrated in Figure 3 below. Firstly, the leadership’s trust in DTs’ relevance based on the leaders’ digitalisation experience influences the leader’s actions towards stakeholders in the digitalisation process. Secondly, leadership’s trustworthiness and ability to build new trust relationships with the different stakeholders is influenced by the leader’s knowledgeability and previous relationships. Hence stakeholders are inclined to trust leaders who are perceived to be credible and knowledgeable. This has a positive impact in effecting desired stakeholders’ trust in the digitalisation process. Thirdly, leadership’s ability to bolster stakeholders’ trust in the digitalisation process reciprocally enables stakeholders’ commitment to involvement for the achievement of digitalisation objectives. Thus, stakeholders’ trust in the digitalisation process extends beyond the process itself to, foremost, the leadership guiding the process.

The leadership influence is enacted through relationships and this trust triality, which is critical to the achievement of digitalisation objectives, as highlighted by the following participants’ assertions:

“Relationships are the foundation of all accomplishments and, sometimes you to go through 3 or 4 meetings to build those relationships and to demonstrate the trust”. [**Participant 14]**

*“It comes down to the chemistry and the credibility of the leader. You can have a credible leader but if he doesn’t have the chemistry and the relationships, it’s not going to happen. So what works in theory doesn’t always work in practice*”. *[****Participant 87]***

“You have to get few people interested and then you go to the others. A very important stakeholder who lived the furthest away, when I called him and I built a relationship with him, when I called him, he said okay, I am going”. [**Participant 03]**

This leadership-digitalisation-stakeholder trust triality construct highlights one specific aspect of the influence of leadership on the involvement of stakeholder involvement in the digitalisation process. The construct demonstrates the interdependencies between its three dimensions and the influence relations thereof. There exists a reciprocal trust enactment cycle among the following six elements: leadership trust in digital technologies, digitalisation’s organisational impact, leadership trustworthiness, drive to involve stakeholders, stakeholders’ commitment to digitalisation, and digitalisation impact on stakeholders. The dynamics of this enactment cycle demonstrate that trust is simultaneously an outcome and an enabler.

### 4.5. Leadership obstructiveness

Finally, there are instances where leadership can be a systemic hinderance to stakeholder involvement. This is likely to occur in two major instances: firstly, when leaders’ interests and stakeholder involvement are divergent, and secondly, when the sector undergoes leadership changes accompanied by structural and stakeholder landscape changes. In the first instance, leadership hierarchical power differences alter the flow in stakeholder involvement processes through systemic tensions between the direct and indirect leadership influence, and the leader’s indirect and direct stakeholder involvement steering abilities and roles. This often materialises in situations where higher level leadership is likely to be totally in control and motivated by personal power interests. This limits manoeuvrability and the effectiveness in involvement efforts consented to by the direct leader and all concerned stakeholders. This has been found to be linked to the higher hierarchical position of the leader and their lack of clinical background. It can have detrimental consequences to stakeholder involvement, digitalisation, healthcare services, and patients’ lives, as reflected in the following participant’s response:

“Some people are interested in power and they told me: “we are in charge, you are not making the decision”. Even though the solution was so obvious to all stakeholders that I was engaging with. Some people value power over purpose and patient and progress. People who actually didn’t have no clinical qualification clearly weren’t confident of doing the job they were supposed to do, for the role of power and gratification unfortunately in healthcare that can cost lives… Clinicians are accountable for the this. I think administrator should also face the same scrutiny as the clinicians…” [**Participant 55]**

This can also be related to systemic issues embedded within the healthcare environment culture where high level hierarchical leaders’ digitalisation priorities and stakeholder involvement prerogative are not aligned. This results in delays throughout the entire digitalisation process in the sector as highlighted by the following participants’ reflections:

“But it’s a complex environment. There is another colleague who did great work that was good for the hospital but who was bullied out of the position because the performance was too good and despite the success of the approach of engaging the different actors. So, even if you have something working and that is better for everybody, sometimes people won’t allow it”. [**Participant 97]**

“They (Leaders) have slowed things down sometimes, due to budget issues and fear of creating worse problems than before. They sometimes take the “if it is not broken, don't fix it approach.” [**Participant 48]**

In the second instance, leadership changes in the healthcare sector can lead to major structural changes in which the speed of implementation of the new leadership’s decisions disrupts the stakeholder landscape and affects ongoing stakeholder involvement actions. This reflects the complexity in the role of leadership, its different levels of influence in stakeholder involvement practice, the existence of systemic and hierarchical barriers to digitalisation leadership’s action, and the implications thereof for effective involvement and digitalisation progress in the sector.

## 5. Discussion

The findings of this study demonstrate the crucial role of leadership in bolstering stakeholder involvement. Leaders must possess the characteristics and traits required for dealing with the complexities of digitalisation in the healthcare sector in the current fast paced innovation environment. Such leaders ought to be visionary, equipped with the necessary competencies, and able to follow a new appropriate approach.^84^ Involvement integrability is achievable when leaders recognise the need to ensure fluidity in digitalisation processes in response to the changing dynamics, and the criticality of stakeholders’ commitment to the achievement of the digitalisation vision. Leadership abilities such as flexibility and responsiveness have been identified as essential in this regard.^85^ While these abilities have equally emerged as critical to stakeholder involvement, this study has particularly revealed the duality of leadership-stakeholder involvement commitment and how this enables effective operationalisation of the digitalisation vision. This is essential in the current digitalisation environment where leadership is essential to fostering organisational commitment for successful of digitalisation.^86^ Moreover, leadership style compatibility and stakeholders’ competencies interdependently contribute to symbiotic interactions among the different healthcare sector stakeholders for improved digitalisation outcomes.^87^ In this regard, Konttila, Siira^88^ highlight the peculiar influence of leadership digital competencies and healthcare professionals’ attributes.

Leadership contributes greatly to stakeholder inclusivity and participation in the digitalisation process by championing engagements in the complex healthcare stakeholder landscape using the necessary engagement strategies, tools and techniques. This is essential in the specific context of the healthcare sector where there exist different types of relations between stakeholders and where different involvement levels and the use of the right communication channels are required to effect the necessary organisational change.^10^ The multiple relationship types between stakeholders in the digitalisation activity system have far reaching implications. The leadership-stakeholder relationship has emerged as being catalytic to effective stakeholder involvement, engagement, and participation. The relationships are particularly influential in effecting trust, a great enabler of DTs’ adoption.^89^ Our findings revealed the interdependencies between leadership and trust enactment in the leadership-digitalisation-stakeholder trust triality of influence. In this multi-directional triangle of influence, leadership trustworthiness and style are determinant in trust relationship building. Trust relations are pivotal for success in multiple stakeholder digitalisation environments.^90^ Moreover, trust functions simultaneously as a precondition and an outcome of effective digitalisation, mediated by leadership that align digital change and stakeholders’ expectations.^91^ On one side, leadership trustworthiness, essential in enacting stakeholder’s trust in the digitalisation process and DTs, encourages stakeholder participation. On the other side, leadership’ trust in the technology increases leadership’s drive to involve stakeholders to achieve success. This highlights the complexity of trust relations in the specific context of stakeholder involvement in digitalisation in the healthcare sector and leadership style’s moderating factor.

Trust is affected by various factors including personal, technological and institutional factors.^92^ Our findings have indicated that leading digital change and involving stakeholders in the complex healthcare environment are intricate interdependent processes where leadership navigate multiple interdependent factors affecting trust building. While transformational and consultative leadership have been identified as appropriate in the healthcare sector,^5^ contextual parameters may require different leadership style to encourage participation. In certain environments, authoritative style may be needed to encourage stakeholder participation and adoption.^87^ Digital leadership’s flexibility in choice of styles and use of hybrid approach is essential in this regard.^93^ Our findings point out the existence of power-interest differences in the sector’s digitalisation’s leadership hierarchy that obstruct stakeholder involvement and the achievement of the digitalisation objectives. Collaborative leadership principles where all actors effectively adjust their power levels for the achievement of a common organisational goal^94^ will alleviate systemic leadership hierarchical barriers and enable good stakeholder involvement in the sector’s digitalisation journey.

## 6. Conclusion

This study reveals the critical role of leadership in stakeholder involvement, and its enabling role in shaping stakeholders’ adherence to the digitalisation vision. The study also highlights leadership influential role in fostering stakeholder participation through a tri-dimensional trust building process in the context of digitalisation in the UK healthcare sector. We emphasise how leadership attributes and leadership approach flexibility are intertwined in facilitating the development of an effective stakeholder involvement environment where stakeholder participation, inclusivity, commitment and DTs’ adoption motivation are achievable. Leadership trustworthiness, knowledgeability, relations, and trust in DTs are pivotal in this multiple-activity system’s dynamics. This study also revealed how interdependencies between leadership hierarchies in the healthcare sector environment, power structures, and personal interests, lead to systemic stakeholder involvement challenges that impact DTs’ adoption. This highlights the sectors’ peculiarities and levels of complexities in comparison to other industries and sectors.

This study’s AT based analysis provides insight into the particularities of the “leader-follower” influence manifestation in a human activity system, where leadership actions in a multi stakeholder environment are mediated by the interaction between multiple sub activities elements, historic and systemic factors and stakeholder involvement practice. The findings contribute to the broad leadership literature highlighting the criticality of leadership relations and leadership’s abilities to build trust relation in creating a functional stakeholder ecosystem in the digitalisation process. The findings contribute to leadership in practice for the achievement of digitalisation objectives by encouraging leaders to be flexible in their approaches and to devise strategies that promote participation, better stakeholder engagements, and trust relations building prioritisation. This study equally contributes to the body of knowledge on trust-building and stakeholder management in the current turbulent digitalisation era where stakeholder involvement is among key digitalisation success enablers. The leadership-digitalisation-stakeholder trust triality concept particularly provides a new perspective on leadership influence’s enactment in a multi-stakeholder environment therefore contributing to the digitalisation leadership and change management body of knowledge.

These findings are likely to be applicable to similar sectors and environments where multiple stakeholders, intra-organisational hierarchies and inter-leadership tensions are likely to affect digitalisation decision-making and digitalisation leadership actions, but where research will be needed to draw definitive conclusions.

### 6.1. Limitations and future work

A limitation of this study is the sole focus of the healthcare sector and the leadership influence manifestation in stakeholder involvement. While these findings are pertinent to similar environments, further research should be undertaken. Firstly, to explore similarities and difference with other industries for generalisability. Secondly, there is a need to understand how leadership navigates systemic barriers to stakeholder involvement. And thirdly, we need to extrapolate trust’s multi-dimensionality and role in digitalisation leadership, stakeholder involvement, and DTs’ adoption across sectors. In line with these, the authors are currently finalising further investigation for the development of a framework of stakeholder involvement for the adoption of DTs from experiences from the healthcare sector and the construction industry of UK.

These findings might be limited by participants’ subjective self-reported leadership perceptions and leadership hierarchy bias which may lead to participants providing desirable or position-aligned responses rather than undistorted views and perspectives.

### 6.2. Declaration

Part of the data set was used for the e-Health 2024 conference publication titled “Enablers of stakeholder involvement in the digitalisation of the UK healthcare sector: an activity theory perspective”.

## Data Availability

The data presented in this study are available on request from the authors. The data are not publicly available because of the ethical clearance obtained.*
